# Associations of serum vitamin B6 status with the risks of cardiovascular, cancer, and all-cause mortality in the elderly

**DOI:** 10.3389/fimmu.2024.1354958

**Published:** 2024-04-18

**Authors:** Pengxi Wang, Jia Huang, Feng Xue, Munire Abuduaini, Yuchang Tao, Hongyan Liu

**Affiliations:** ^1^ Department of Medical Genetics, Henan Provincial People’s Hospital, People’s Hospital of Zhengzhou University, Zhengzhou, Henan, China; ^2^ College of Public Health, Zhengzhou University, Zhengzhou, China

**Keywords:** serum vitamin B6, pyridoxal 5’-phosphate (PLP), 4-pyridoxal acid (4-PA), mortality, elderly

## Abstract

**Background:**

There are few studies investigating the relationship between serum vitamin B6 and mortality risk in the elderly. This study hereby evaluated the associations between biomarkers of serum vitamin B6 status and cardiovascular, cancer, and all-cause mortality risks in the elderly.

**Methods:**

Our study included a total of 4,881 participants aged 60 years or older from the National Health and Nutrition Examination Survey (NHANES) 2005-2010. Serum vitamin B6 status was estimated based on levels of pyridoxal 5’-phosphate (PLP), 4-pyridoxic acid (4-PA), and vitamin B6 turnover rate (4-PA/PLP) detected by high-performance liquid chromatography. Survival status and corresponding causes of death were matched through the National Death Index records through December 31, 2019. Multivariate Cox regression model was adopted to assess the relationships between serum vitamin B6 status and the risk of mortality.

**Results:**

During a median follow-up period of 10.33 years, 507 cardiovascular deaths, 426 cancer deaths, and 1995 all-cause deaths were recorded, respectively. In the multivariate-adjusted Cox model, the hazard ratios (HRs) and 95% confidence intervals (CIs) for the highest versus the lowest quartiles of PLP, 4-PA, and 4-PA/PLP were 0.70(0.54-0.90), 1.33(0.88-2.02), and 2.01(1.41-2.79) for cardiovascular mortality, 0.73(0.52-1.02), 1.05(0.71-1.57), and 1.95(1.25-3.05) for cancer mortality, and 0.62(0.53-0.74), 1.05(0.82-1.34), and 2.29(1.87-2.79) for all-cause mortality, respectively.

**Conclusion:**

Our study found that lower serum PLP levels were associated with increased risks of cardiovascular and all-cause mortality among the elderly population. And higher vitamin B6 turnover rate was associated with increased risks of cardiovascular, cancer, and all-cause mortality.

## 1 Introduction

The World Health Organization (WHO) demographic survey shows that both the number and proportion of the world’s elderly population are increasing at an unprecedented speed, with a projected global population of 2.1 billion people aged 60 or over by 2050 (1). Aging increases the risk of malnutrition due to physiological changes, physical and cognitive decline, social and environmental influences, and multiple diseases (2). Malnutrition not only extensively affects the quality of life for older adults, but also increases their risks of morbidity and mortality (3). Therefore, identifying modifiable factors that can change the nutritional status of the elderly is critical for maintaining their health and preventing or delaying premature death.

Vitamin B6, an important nutrient, exists in the human body in six interchangeable forms: pyridoxal (PL), pyridoxine (PN), pyridoxamine (PM), and their respective phosphorylated derivatives (4, 5). Among them, pyridoxal 5’-phosphate (PLP), as a coenzyme form of vitamin B6, is involved in the catalysis of more than 160 different functions (4, 6). 4-Pyridoxine (4-PA) is the major metabolite of vitamin B6, and approximately 40-60% of dietary vitamin B6 is excreted in this form (5). Currently, PLP, 4-PA, and 4-PA/(PLP +PL) (PAr) are the most common direct biomarkers for reflecting vitamin B6 status (6).

Vitamin B6 participates in many biochemical reactions involving amino acid substrates (4). For example, it affects homocysteine metabolism by influencing the activities of cystathionine-β-synthase (CBS) and γ-cystathionase (7). In addition, Vitamin B6 also affects immune function, inflammation, antioxidants, and cellular signaling (8, 9). Currently, several epidemiological studies have demonstrated that higher levels of vitamin B6 are associated with a reduced risk of cardiovascular disease (10), cancer (11), and all-cause mortality (12, 13). Studies have indicated a high prevalence of vitamin B6 deficiency among individuals diagnosed with type 2 diabetes (14). However, most of these studies investigated the relationship of vitamin B6 levels with mortality in patients with specific diseases (15–18). Limited evidence is available regarding the health effects of vitamin B6 among the elderly. To fill this research gap and explore whether vitamin B6 may prevent or delay premature death in older adults, we conducted a prospective study to assess the relationships between serum vitamin B6 levels and the risks of cardiovascular, cancer, and all-cause mortality in the U.S. elderly.

## 2 Materials and methods

### 2.1 Study population

**Figure 1:**
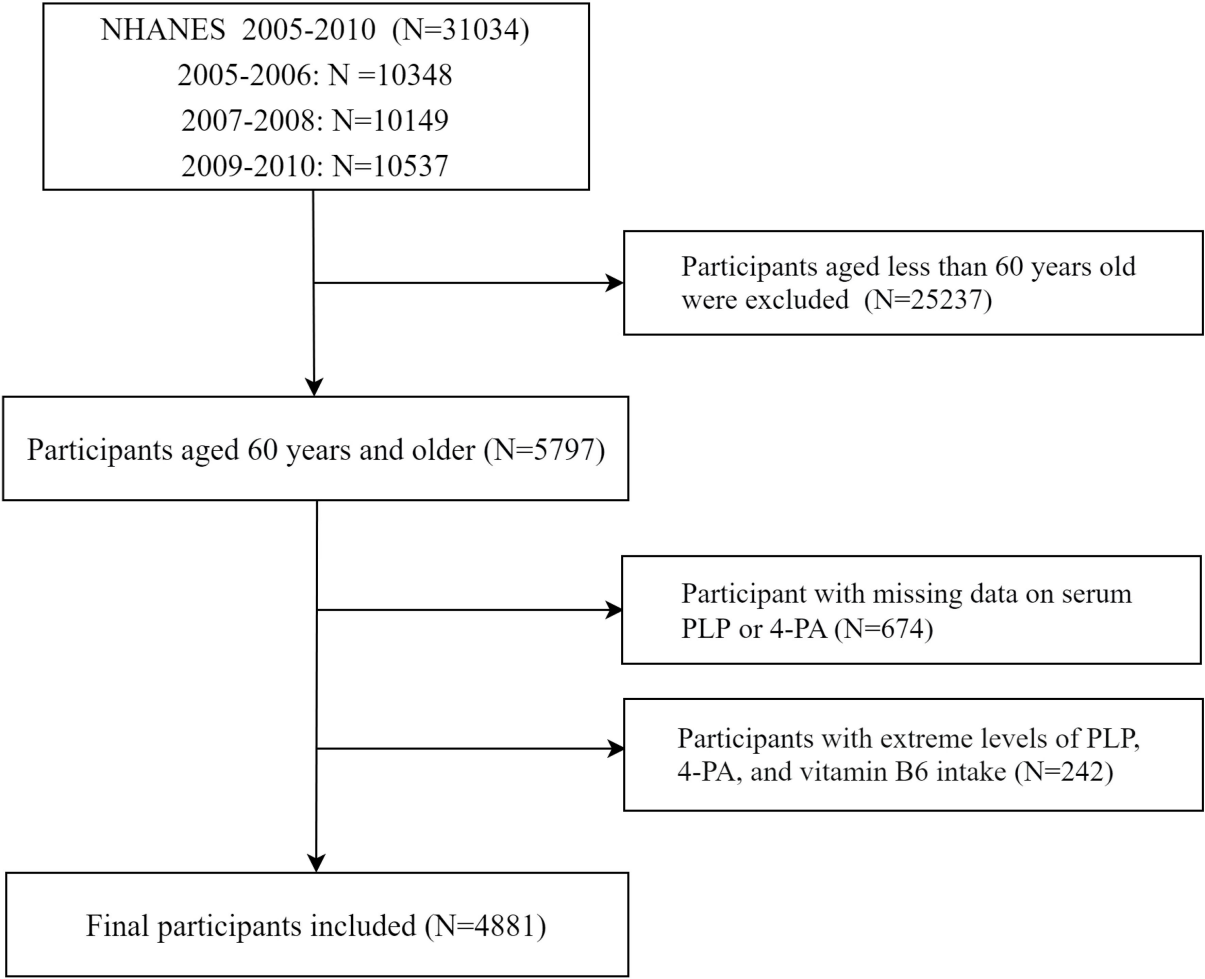
Flowchart of participant selection.

The National Health and Nutrition Examination Survey (NHANES) is a nationally representative survey that assesses the health and nutrition status of the U.S. civilian population through personal interviews, laboratory tests, and standardized medical examinations (19, 20). The survey begins by randomly selecting counties in the United States, then randomly selecting several neighborhoods from each county, followed by the random selection of a number of families from each neighborhood, and finally randomly selecting family members based on age, gender, and race. The survey is conducted every two years. Considering the complex survey design used for NHANES, which includes oversampling, stratification, and clustering, the data were weighted prior to data analysis according to the NHANES analysis guidelines (21). The concentration of vitamin B6 was measured from 2003 to 2010 in four cycles of NHANES. However, measurements of vitamin B6 levels from 2005-2010 could not be compared with those from 2003-2004 due to the lack of available adjustments. This study included three NHANES cycles from 2005 to 2010. Participants with the following characteristics were excluded from our study: a) Younger than 60 years of age at the initial survey; b) Missing data for PLP, 4-PA, or mortality outcomes; c) Presence of outliers on PLP, 4-PA and vitamin B6 according to z-core test. As indicated in **Figure 1**, a total of 4,881 participants were finally included. All participants have received written informed consent.

### 2.2 Measurement of vitamin B6 status

Serum PLP (nmol/L) and 4-PA (nmol/L) concentrations were measured to estimate vitamin B6 levels. The serum PLP and 4-PA concentrations of NHANES participants were determined using high-performance liquid chromatography. Additionally, since there was no available serum concentration of PL, we used 4-PA/PLP instead of 4-PA/(PL+ PLP) to evaluate the turnover rate of vitamin B6. This is because the intraclass correlation coefficient (ICC) for 4-PA/PLP is similar to that of 4-PA/(PL+PLP) (22), at least among non-users of vitamin B6 supplements, and PLP has a strong association with PL (23). Therefore, the use of 4-PA/PLP can be considered as an effective surrogate for assessing vitamin B6 turnover.

### 2.3 Ascertainment of mortality

Survival information was obtained from the National Center for Health Statistics by matching NHANES participants with the probability of a National Death Index (NDI) record through December 31, 2019. The NDI includes information on death records of individuals who have died in the United States since 1979, and their survival status as well as the corresponding detailed causes of death are recorded annually. The disease-specific deaths were determined by the 10th revision of the International Classification of Diseases (ICD-10). The primary outcomes of our study were cardiovascular, cancer, and all-cause mortality. Cardiovascular mortality was defined as the ICD-10 codes for I00-I09, I11, I13, I20-I51, or I60-I69. Cancer mortality was defined as ICD-10 codes for C00-C97.

### 2.4 Covariates

Based on reviewing relevant literature (17, 24), we selected the following covariates in the model: age (<70, 70-80 or ≥80 years), sex (male or female), drink (yes or no), smoke (never or former or current), education (Less than high school, High school or equivalent, and College or above), race ethnicity (Hispanic, non-Hispanic white, non-Hispanic black, and Race-including multi-racial), physical activity (low, moderate, or vigorous), cholesterol (≥6.2 mmol/L or <6.2 mmol/L), and self-reported physician diagnosed cardiovascular disease (yes or no), cancer (yes or no), hypertension (yes or no) and type 2 diabetes (yes or no). Body mass index (BMI) was calculated as weight (kg) divided by height (meters squared). Moreover, Vitamin B6 intake (mg/day) was assessed by trained researcher using 24-hour dietary recall.

### 2.5 Statistical analysis

Continuous variables that conform to a normal distribution, as determined by the Shapiro-Wilk test, are described using the survey-weighted mean (standard deviation, SD). Otherwise, they are described using the survey-weighted median (interquartile range, IQR). Categorical variables are presented as numbers and survey-weighted percentages. The Chi-square test or Wilcoxon rank sum test was used for the different analysis of survival status for categorical variables and continuous variables with skewed distribution. The t-test was used for continuous variables with a normal distribution. Multiple imputation was used to impute these covariates with missing values (25).

The survey-weighted Cox regression model was used to assess the relationships of PLP, 4-PA, and 4-PA/PLP with cardiovascular, cancer, and all-cause mortality. Biomarkers of serum vitamin B6 (i.e., PLP, 4-PA, and 4-PA/PLP) were categorized into quartiles, with the first quartile as the reference group. Three models were adopted: The crude model was not adjusted anything. Model 1 included adjustment for sex and age. Model 2 included adjustments for the variables in model 1 as well as for smoke, drink, ethnicity, education, physical activity and BMI. Model 2 was considered as the main model. We conducted survival analyses using covariate-adjusted Kaplan-Meier curves to explore the effects of 4-PA, PLP, and 4-PA/PLP quartiles on survival time in older adults; we also tested for non-linear associations between biomarkers of vitamin B6 status (log2 transformed) and the risk of mortality using restricted cubic splines (RCS), with four knots at percentiles 5, 35, 65, and 95.

We conducted stratified analyses by age (<70 or ≥70 years), sex (male or female), BMI (<30 kg/m^2^ or ≥30 kg/m^2^), smoke (never or former or current), and drink (yes or no) to examine whether these relationships were modified by these potential modifiers. We explored the interactions of PLP, 4-PA, and 4-PA/PLP with the modifier by adding a multiplicative interaction term (i.e., quartiles PLP, 4-PA, or 4-PA/PLP parameter * potential modifiers).

To examine the robustness of the result, two sensitivity analyses were performed: (1) further adjustment for common chronic diseases and dietary vitamin B6 intake based on model 2; (2) excluding patients who died within 2 years of follow-up.

All statistical analyses and graphs were performed using R version 4.3.1 (‘survey’, ‘plotRCS’, and ‘gtsummary’ packages), and two-sided P values <0.05 was deemed to be statistically significant.

## 3 Results

### 3.1 General characteristics

A total of 4881 participants participated in our study, with a mean age of 70.70 (SD: 7.32) years, including 2,440 males and 2441 females. The median (IQR) concentrations of serum PLP, 4-PA, and 4-PA/PLP were 39.8 (56.1) nmol/L, 35.1 (53.9) nmol/L, and 0.9 (0.9), respectively. During a median (IQR) follow-up of 10.33 (4.8) years, 507(10.4%) cardiovascular deaths, 426 (8.7%) cancer deaths and 1995 (40.9%) all-cause deaths were recorded, respectively. The general characteristics of participants are shown in **Table 1**. In general, participants who survive are younger, more likely to be female, never smokers, Non-Hispanic White, take more physical activity, have a higher level of education, intake of more vitamin B6, and less likely to have hypertension, diabetes, cancer, and cardiovascular disease.

**Table 1:** Baseline characteristics of the study population.

Characteristic	Total	Survivors	Death	P _trend_
	N= 4881	N= 2886 (61%)	N= 1995 (39%)	
Age, year*^##^				<0.001
<70	2,289 (50%)	1,787 (65%)	502 (27%)	
70-80	1,613 (32%)	887 (28%)	726 (37%)	
≥80	979 (18%)	212 (6.5%)	767 (36%)	
Male*^#^	2,440 (45%)	1,316 (43%)	1,124 (48%)	<0.001
Smoke*^#^				<0.001
never	2,307 (47%)	1,492 (51%)	815 (42%)	
former or current	2,574 (53%)	1,394 (49%)	1,180 (58%)	
Drink*^#^				0.028
NO	1,796 (35%)	1,064 (33%)	732 (37%)	
YES	3,085 (65%)	1,822 (67%)	1,263 (63%)	
**Race** *^#^				0.002
Hispanic	1,037 (6.7%)	782 (8.0%)	255 (4.6%)	
Non-Hispanic White	2,785 (81%)	1,458 (79%)	1,327 (84%)	
Non-Hispanic Black	909 (8.4%)	547 (8.2%)	362 (8.7%)	
other	150 (4.0%)	99 (4.6%)	51 (3.1%)	
Education*^##^				<0.001
Less than high school	1,757 (25%)	955 (20%)	802 (33%)	
high school/GED	1,203 (27%)	702 (27%)	501 (28%)	
College or above	1,921 (48%)	1,229 (53%)	692 (39%)	
**Physical activity** *^##^				<0.001
Low	2,414 (43%)	1,223 (35%)	1,191 (56%)	
moderate	742 (17%)	558 (22%)	184 (10%)	
vigorous	1,725 (39%)	1,105 (43%)	620 (34%)	
Hypertension*^#^	2,911 (58%)	1,643 (55%)	1,268 (64%)	<0.001
Diabetes*^#^	1,104 (19%)	580 (16%)	524 (24%)	<0.001
cholesterol*^#^	763 (16%)	491 (18%)	272 (14%)	<0.001
CVD*^#^	1,242 (24%)	497(16.4%)	794(38%)	<0.001
cancer*^#^	954 (22%)	448 (18%)	506 (27%)	<0.001
**Vitamin B6 intake**, mg/d**^##^	3.25 (2.28)	3.29 (2.23)	3.16 (2.29)	0.003
**BMI**, kg/m^2^**^##^	28.08 (7.22)	28.37 (7.14)	27.54 (7.42)	<0.001

^*^Data were presented as numbers (percentages).

^**^Data were presented as median (interquartile range, IQR).

Chi-square test was used for data.

^#^Wilcoxon rank sum test was used for data.

Shapiro-Wilk test: P _vitamin B6 intake_ <0.001; P _BMI_ <0.001.

PIR, family income-to-poverty ratio; BMI, body mass index; CVD, Cardiovascular disease.

### 3.2 Serum vitamin B6 status with cardiovascular, cancer, and all-cause mortality

**Figure 2:**
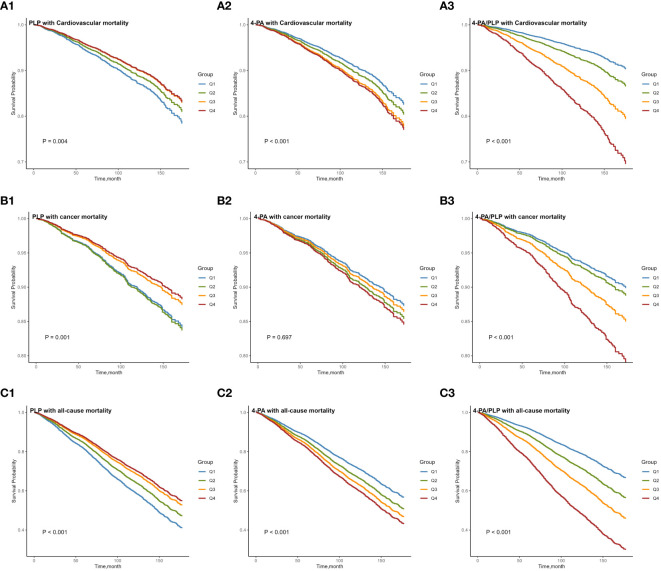
Kaplan-Meier survival curves for CVD **(A)**, cancer **(B)**, and all-cause **(C)** mortality in U.S. older adults grouped by quartiles of serum vitamin B6 biomarkers. Adjusted for age, sex, smoke, drink, race/ethnicity, education, activity and BMI. BMI, body mass index; CVD, cardiovascular disease; PLP, pyridoxal-5’-phosphate; 4-PA, 4-pyridoxic acid.

As shown in **Tables 2**, **3**, serum PLP concentrations were negatively associated with cardiovascular and all-cause mortality. Additionally, serum PLP concentrations were negatively associated with cancer mortality when adjusted for age and sex (model 1, P_Q4vs.Q1 =_ 0.002), but this statistical association disappeared when adjusted for sociodemographic and lifestyle factors (model 2). No statistical association was observed between serum 4-PA concentrations and the risk of mortality. Furthermore, the vitamin B6 turnover rate, 4-PA/PLP, showed a significant positive association with the risks of cardiovascular, cancer, and all-cause mortality. After adjusting for sociodemographic and lifestyle factors in a multivariate model (model 2), the weighted Cox regression results indicate that the hazard ratios (HRs) and 95% confidence intervals (CIs) for the highest versus the lowest quartiles of PLP, 4-PA, and 4-PA/PLP were 0.70(0.54-0.90), 1.33(0.88-2.02), and 2.01(1.41-2.79) for cardiovascular mortality, 0.73(0.52-1.02), 1.05(0.71-1.57), and 1.95(1.25-3.05) for cancer mortality, and 0.62(0.53-0.74), 1.05(0.82-1.34), and 2.29(1.87-2.79) for all-cause mortality, respectively. The results of the covariate-adjusted Kaplan-Meier survival curves (**Figure 2**) indicate that low levels of PLP and high levels of 4-PA and 4-PA/PLP were associated with higher probability of death, except for the association between 4-PA and cancer death (P=0.697). This is consistent with the results of the crude model of Cox regression and consistent with the effect direction of the model 2, although some associations in model 2 did not reach significance.

**Table 2:** Hazard ratio (HRs) and 95% CIs for cardiovascular mortality across quartiles of vitamin B6 biomarkers among the U.S. older adults (N=4881; CVD-deceased = 507).

	Quartile of vitamin B6 biomarkers							
	Quintile1	Quintile2	P-value	Quintile3	P-value	Quintile4	P-value	
PLP, nmol/L								
Range	<23.0	23-39.8		39.8-74.6		>74.6		
Median (IQR)	23.0(17.6)	30.7(8.7)		54.6(16.5)		118.0(79.1)		
No. deaths/total	145/1220	127/1219		116/1221		119/1221		
Crude Model	Ref	0.83(0.62-1.12)	0.230	0.74(0.54-1.02)	0.064	0.61(0.48-0.78)	<0.001	
Model 1	Ref	0.79(0.58-1.05)	0.108	0.68(0.50-0.92)	0.013	0.59(0.46-0.76)	<0.001	
Model 2	Ref	0.83(0.62-1.11)	0.216	0.77(0.56-1.05)	0.096	0.70(0.54-0.90)	0.005	
4-PA, nmol/L								
Range	<20.6	20.6-35.1		35.1-74.5		>74.5		
Median (IQR)	15.3(5.7)	26.8(7.2)		48.2(17.4)		138.0(123.1)		
No. deaths/total	80/1217	118/1221		146/1222		163/1221		
Crude Model	Ref	1.45(0.95-2.23)	0.088	1.59(1.00-2.53)	0.048	1.84(1.27-2.65)	0.003	
Model 1	Ref	1.03(0.68-1.56)	0.896	1.10(0.69-1.74)	0.699	1.15(0.77-1.71)	0.406	
Model 2	Ref	1.13(0.74-1.71)	0.573	1.24(0.76-2.01)	0.391	1.33(0.88-2.02)	0.178	
Ratio 4-PA/PLP								
Range	<0.6	0.6-0.9		0.9-1.5		>1.5		
Median (IQR)	0.48(0.16)	0.76(0.14)		1.15(0.27)		2.15(1.25)		
No. deaths/total	62/1221	90/1220		149/1218		206/1222		
Crude Model	Ref	1.50(1.05-2.13)	0.024	2.23(1.67-2.97)	<0.001	3.94(2.87-5.42)	<0.001	
Model 1	Ref	1.22(0.85-1.77)	0.279	1.59(1.18-2.14)	0.002	2.26(1.65-3.09)	<0.001	
Model 2	Ref	1.20(0.82,1.75)	0.343	1.48(1.09,2.01)	0.013	2.01(1.41-2.79)	<0.001	

Crude Model: did not adjust anything.

Model 1: adjusted for age and sex.

Model 2: further adjusted for smoke, drink, ethnicity, education, activity and BMI.

IQR, interquartile range; BMI, body mass index; PLP, pyridoxal-5’-phosphate; 4-PA, 4-pyridoxic acid.

**Table 3:** Hazard ratio (HRs) and 95% CIs for cancer mortality and all-cause mortality across quartiles of vitamin B6 biomarkers among the U.S. older adults (N=4881; Cancer-deceased =426; All-cause deceased =1995).

	Quartile of vitamin B6 biomarkers						
	Quintile1	Quintile2	P-value	Quintile3	P-value	Quintile4	P-value
Cancer mortality							
PLP, nmol/L							
No. deaths/total	118/1220	122/1219		98/1221		88/1221	
Crude Model	Ref	1.08(0.80-1.44)	0.630	0.83(0.59-1.18)	0.306	0.61(0.45-0.84)	0.002
Model 1	Ref	1.04(0.78-1.39)	0.796	0.78(0.56-1.11)	0.169	0.61(0.44-0.83)	0.002
Model 2	Ref	1.12(0.84-1.50)	0.444	0.87(0.61-1.23)	0.425	0.73(0.52-1.02)	0.068
4-PA, nmol/L							
No. deaths/total	102/1217	111/1221		103/1222		110/1221	
Crude Model	Ref	1.12(0.83-1.52)	0.456	1.06(0.76-1.47)	0.745	1.09(0.80-1.48)	0.598
Model 1	Ref	0.97(0.71-1.32)	0.835	0.89(0.64-1.25)	0.510	0.90(0.64-1.26)	0.528
Model 2	Ref	1.06(0.76-1.47)	0.744	1.00(0.70-1.44)	0.989	1.05(0.71-1.57)	0.795
Ratio 4-PA/PLP							
No. deaths/total	79/1221	88/1220		117/1218		142/1222	
Crude Model	Ref	1.42(0.97-2.08)	0.075	1.66(1.06-2.58)	0.026	2.63(1.76-3.94)	<0.001
Model 1	Ref	1.27(0.87-1.87)	0.212	1.42(0.89-2.28)	0.144	2.10(1.36-3.25)	<0.001
Model 2	Ref	1.26(0.86-1.85)	0.244	1.36(0.85-2.20)	0.202	1.95(1.25-3.05)	0.003
All-cause mortality							
PLP, nmol/L							
No. deaths/total	605/1220	511/1219		449/1221		430/1221	
Crude Model	Ref	0.82(0.71-0.94)	0.006	0.67(0.58-0.77)	<0.001	0.56(0.47-0.66)	<0.001
Model 1	Ref	0.78(0.68-0.89)	<0.001	0.62(0.54-0.71)	<0.001	0.54(0.46-0.64)	<0.001
Model 2	Ref	0.82(0.72-0.95)	0.006	0.68(0.58-0.78)	<0.001	0.62(0.53-0.74)	<0.001
4-PA, nmol/L							
No. deaths/total	400/1217	474/1221		537/1222		584/1221	
Crude Model	Ref	1.11(0.93-1.32)	0.245	1.30(1.08-1.57)	0.006	1.34(1.10-1.62)	0.003
Model 1	Ref	0.84(0.71-0.99)	0.033	0.96(0.79-1.17)	0.704	0.91(0.74-1.13)	0.406
Model 2	Ref	0.92(0.76-1.11)	0.384	1.07(0.87-1.31)	0.514	1.05(0.82-1.34)	0.688
Ratio 4-PA/PLP							
No. deaths/total	276/1221	397/1220		549/1218		773/1222	
Crude Model	Ref	1.63(1.34-1.99)	<0.001	2.25(1.85-2.72)	<0.001	3.85(3.16-4.69)	<0.001
Model 1	Ref	1.40(1.15-1.69)	<0.001	1.73(1.45-2.07)	<0.001	2.49(2.08-2.98)	<0.001
Model 2	Ref	1.39(1.14-1.69)	0.001	1.67(1.37-2.03)	<0.001	2.29(1.87-2.79)	<0.001

Crude Model: did not adjust anything.

Model 1: adjusted for age and sex.

Model 2: further adjusted for smoke, drink, ethnicity, education, activity and BMI.

BMI, body mass index; PLP, pyridoxal-5’-phosphate; 4-PA, 4-pyridoxic acid.

**Figure 3:**
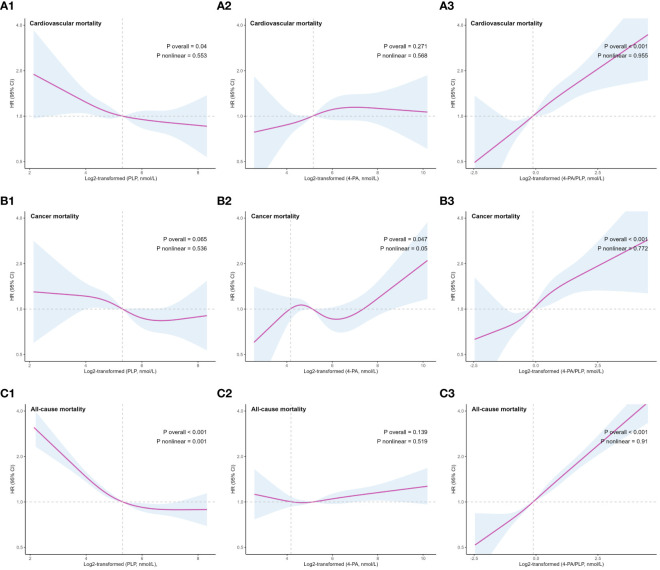
Restricted cubic splines (5th, 35th, 65th, 95th nodes) for associations of serum vitamin B6 biomarkers and CVD **(A)**, cancer **(B)**, and all-cause **(C)** mortality in U.S. older adults. Adjusted for age, sex, smoke, drink, race/ethnicity, education, activity and BMI. BMI, body mass index; CVD, cardiovascular disease; PLP, pyridoxal-5’-phosphate; 4-PA, 4-pyridoxic acid.

In further analyses, adjusted RCS models were used to describe the dose-response relationships between vitamin B6 and the risks of cardiovascular, cancer, and all-cause mortality (**Figure 3**). We observed significant associations between PLP levels and the risks of cardiovascular (P=0.040) and all-cause (P<0.001) mortality, 4-PA levels and the risk of cancer mortality (P=0.047), and 4-PA/PLP ratio and risks of cardiovascular, cancer, and all-cause mortality (P<0.001 for overall association). No statistically significant nonlinear relationship was found between vitamin B6 biomarkers and the risk of mortality, except for an L-shaped nonlinear relationship between serum PLP and all-cause mortality (P = 0.001) with an inflection point at 39.54 nmol/L.

### 3.3 Stratified and sensitivity analyses

The results of subgroup analyses are presented in **Supplementary Tables S1-S3**. The direction of the associations between biomarkers of vitamin B6 status and mortality was consistent across subgroups, although in some subgroups the association did not reach statistical significance. Statistically significant interactions existed on some factors. The inverse association between serum PLP concentration and all-cause mortality was more significant in individuals aged ≥70 years, and the inverse association between serum PLP concentration and cardiovascular mortality was also more significant in the male population. The positive association between 4-PA/PLP and cancer mortality was stronger among drinkers, males, and individuals aged < 70 years. In addition, sensitivity analyses showed that the associations of serum PLP levels with cardiovascular and all-cause mortality, and the associations of 4-PA/PLP with cardiovascular, cancer, and all-cause mortality remained significant after further adjustments for common chronic diseases and dietary vitamin B6 intake or exclusion of participants who died within 2 years of follow-up, as shown in **Tables 4**, **5**.

**Table 4:** Hazard ratio (HRs) and 95% CIs for mortality across quartiles of vitamin B6 biomarkers after further adjustment for common chronic diseases and dietary vitamin B6 intake.

	Quartile of vitamin B6 biomarkers						
	Quintile1	Quintile2	P-value	Quintile3	P-value	Quintile4	P-value
CVD mortality							
PLP	Ref	0.83(0.61-1.13)	0.237	0.80(0.59-1.10)	0.168	0.72(0.57-0.91)	0.007
4-PA	Ref	1.07(0.73-1.58)	0.726	1.19(0.77-1.84)	0.426	1.25(0.85-1.83)	0.264
Ratio 4-PA/PLP	Ref	1.18(0.81-1.71)	0.383	1.36(1.01-1.83)	0.040	1.74(1.27-2.39)	<0.001
Cancer mortality							
PLP	Ref	1.11(0.83-1.48)	0.476	0.84(0.58-1.21)	0.342	0.70(0.50-0.99)	0.045
4-PA	Ref	1.01(0.74-1.38)	0.938	0.93(0.64-1.36)	0.717	0.99(0.67-1.46)	0.946
Ratio 4-PA/PLP	Ref	1.24(0.84-1.82)	0.285	1.33(0.81-2.17)	0.260	1.91(1.23-3.98)	0.004
All-cause mortality							
PLP	Ref	0.83(0.73-0.95)	0.005	0.70(0.60-0.82)	<0.001	0.64(0.54-0.75)	<0.001
4-PA	Ref	0.89(0.79-1.05)	0.157	1.04(0.85-1.26)	0.714	1.00(0.79-1.26)	0.971
Ratio 4-PA/PLP	Ref	1.35(1.10-1.67)	0.005	1.57(1.30-1.90)	<0.001	2.05(1.69-2.50)	<0.001

Adjusted for age, sex, smoke, drink, race/ethnicity, education, activity, BMI, vitamin B6 intake, hypertension, diabetes, cholesterol, CVD, cancer.

BMI, body mass index; CVD, cardiovascular disease; PLP, pyridoxal-5’-phosphate; 4-PA, 4-pyridoxic acid.

**Table 5:** Hazard ratio (HRs) and 95% CIs for mortality across quartiles of vitamin B6 biomarkers after excluding participants who died within 2 years of follow-up (N=4640).

	Quartile of vitamin B6 biomarkers						
	Quintile1	Quintile2	P-value	Quintile3	P-value	Quintile4	P-value
CVD mortality							
PLP	Ref	0.90(0.68-1.20)	0.484	0.84(0.59-1.20)	0.336	0.74(0.57-0.98)	0.032
4-PA	Ref	1.16(0.74-1.81)	0.512	1.38(0.84-2.28)	0.204	1.42(0.90-2.26)	0.132
Ratio 4-PA/PLP	Ref	1.12(0.74-1.69)	0.596	1.44(1.05-1.98)	0.025	1.92(1.38-2.67)	<0.001
Cancer mortality							
PLP	Ref	1.29(0.90-1.83)	0.164	1.02(0.69-1.49)	0.938	0.91(0.63-1.32)	0.625
4-PA	Ref	1.07(0.73-1.57)	0.740	1.02(0.67-1.55)	0.922	1.12(0.72-1.74)	0.615
Ratio 4-PA/PLP	Ref	1.30(0.88-1.93)	0.188	1.33(0.78-2.28)	0.294	1.99(1.26-3.15)	0.003
All-cause mortality							
PLP	Ref	0.91(0.78-1.06)	0.232	0.73(0.62-0.87)	<0.001	0.70(0.62-0.87)	<0.001
4-PA	Ref	0.93(0.77-1.12)	0.452	1.10(0.88-1.38)	0.385	1.09(0.82-1.44)	0.553
Ratio 4-PA/PLP	Ref	1.36(1.13-1.63)	0.001	1.65(1.37-1.99)	<0.001	2.12(1.74-2.58)	<0.001

Adjusted for age, sex, smoke, drink, race/ethnicity, education, activity, BMI, vitamin B6 intake, hypertension, diabetes, cholesterol, CVD, cancer.

BMI, body mass index; CVD, cardiovascular disease; PLP, pyridoxal-5’-phosphate; 4-PA, 4-pyridoxic acid.

## 4 Discussion

Based on the analysis of NHANES data, we observed inverse association between serum PLP levels and the risks of cardiovascular as well as all-cause mortality. No significant associations were found between serum 4-PA levels and the risk of mortality. Furthermore, there was positive associations between 4-PA/PLP and the risks of cardiovascular, cancer, and all-cause mortality. After a series of sensitivity analyses, the results were robust.

PLP levels are an effective marker for assessing vitamin B6 levels (6). The inverse relationships of serum PLP concentration with all-cause and cardiovascular mortality is consistent with several previous investigations. For instance, Isidor Minovi´c et al. observed in the Prevention of Renal and Vascular End-stage Disease study that lower plasma PLP concentrations were associated with higher cardiovascular disease morbidity and mortality (10). Studies from European cohorts showed that study participants with higher circulating levels of vitamin B6 had a lower risk of developing renal cell carcinoma and a higher survival rate after diagnosis (26). Pusceddu et al. found that vitamin B6 deficiency was a risk factor for all-cause mortality in the Ludwigshafen Risk and Cardiovascular Health Study (27). Paula Schorgg et al. observed that high PLP levels were associated with reduced cardiovascular mortality among individuals aged ≥65 years, but not in the broader population (28). However, a study from Caerphilly showed that vitamin B6 levels were associated with non-cardiovascular disease mortality in men, but not with cardiovascular or cancer mortality (29). Another study conducted on the general population also observed a negative association between PLP and all-cause mortality, but found no association with cardiovascular or cancer mortality (24). These discrepancies may be due to the different study populations. With aging, the aorta stiffens due to increased collagen and decreased elastin, thereby increasing the risk of cardiovascular disease (30, 31). This phenomenon might amplify the impact of vitamin B6 on the risk of cardiovascular death.

In addition to PLP levels, we also investigated the health effects of serum 4-PA levels and 4-PA/PLP on mortality. 4-PA is a catabolized vitamin B6 produced by PL in the liver and has a high clearance rate in kidney (32). 4-PA/PLP instead of PAr was used as an indicator of vitamin B6 turnover, indicating low vitamin B6 status owing to altered tissue distribution or enhanced vitamin B6 turnover (22). Interestingly, our results suggested that 4-PA/PLP was significantly and positively associated with cardiovascular, cancer, and all-cause mortality, but 4-PA was not associated with mortality risk. Cohort study from Norway indicated that plasma PAr is a good predictor of all-cause mortality risk in patients with coronary artery disease (33). The Hordaland Health Study (HUSK) showed that higher PAr in general population was associated with increased overall cancer risk (34). Qianwei Cui et al. observed inverse associations between vitamin B6 conversion rates and the risk of cardiovascular and all-cause mortality in hypertensive adults (35). These studies support our findings. However, Qianwei Cui et al. also suggested that higher levels of 4-PA were associated with an increased risk of all-cause mortality in hypertensive adults. Dandan Zhang et al. observed a positive relationship between serum 4-PA levels and all-cause mortality in individuals with T2DM (17). These differences may be attributed to the study population and methods. Our unweighted analysis of the NHANES data also showed a positive association between 4-PA and all-cause mortality, but this association disappeared after conducting weighted analysis. According to the NHANES analysis guidelines, the results of the weighted analysis are more reliable. Furthermore, Paula Schorgg et al. observed that 4-PA/PLP was associated with cancer and all-cause mortality in the elderly population, but not with cardiovascular mortality (28). They also noted that no statistical association was found between 4-PA and the mortality risk. This discrepancy may be attributed to their adjustment for variables related to inflammation. The impact of vitamin B6 on risk mortality may be associated with inflammatory processes, and potential collinearity among variables when adjusting models for inflammatory factors could reduce the predictive power of vitamin B6 for mortality risk.

The association of vitamin B6 levels with the risk of mortality may be mediated by its involvement in immune and inflammatory processes. Studies have demonstrated that pyridoxine supplementation significantly enhances the immune response in the elderly individuals (36), patients with renal failure (37), and critically ill patients (38). In the elderly, vitamin B6 depletion severely affects lymphocyte count, mitogenic response of T and B cell mitogens, and interleukin-2 production, and these immune markers return to normal with vitamin B-6 supplementation (39). In recent years, vitamin B6-dependent inflammatory pathways have been extensively studied. Previous studies have demonstrated a negative association between plasma PLP levels and several inflammatory markers (40, 41), such as the acute-phase markers c-reactive protein (CRP) and the kynurenine/tryptophan ratio (KTR). The regulation of PLP-dependent enzymes and associated pathways in the inflammatory response is influenced by the distribution of vitamin B6 in tissues (42). During inflammation, depletion of tissue-specific vitamin B6 leads to low plasma levels of PLP (43). Some of the increases in 4-PA/PLP levels may be attributed to the elevated demand for PLP in tissues, and 4-PA/PLP corrects for potential confounders that affect 4-PA and PLP proportionally, such as supplement intake, contributing to the robustness and specificity of the 4-PA/PLP. In addition, several publications have confirmed that systemic inflammation is strongly associated with cardiovascular disease development (44, 45). The causal relationship between chronic inflammation and cancer development has also been extensively studied (46). Chronic inflammation can cause cellular DNA mutation by inducing oxidative and nitrosative stress and can also promote cancer development by affecting tissue repair, genotoxic stability, invasion, proliferative response, and metastasis (46–48). Thus, the adverse effects of insufficient circulating vitamin B6 on inflammatory responses and immune processes may increase the risk of premature death.

Previous investigations have focused on the relationship between vitamin B6 and mortality in disease-specific populations, with limited evidence from studies in older populations. In this study, we used a complex, stratified, multistage probabilistic sampling method to obtain a sample. Vitamin B6 status was expressed according to objectively measured serum parameters and adjusted for various potential confounders in the Cox regression model. This makes our estimates more robust and accurate. Several limitations are also present in our study. First, our study was observational. Future trials and experimental studies are still needed to verify these findings and explore the underlying mechanisms. Second, we only had data measured at baseline and lacked interview data from multiple periods. Third, family history of chronic disease was considered a common confounder for which survey data is lacking. In our sensitivity analysis, we further adjusted for prevalent chronic diseases to account for the potential impact of family history on the results. Fourth, serum concentrations of the vitamin B6 biomarker pyridoxal (PL) and 4-PA/(PLP+PL) (PAr) could not be obtained because our study was retrospective. However, 4-PA/PLP has a similar intraclass correlation coefficient (ICC) to 4-PA/(PL+ PLP) (22) and PLP has a strong association with PL (23).

## 5 Conclusions

In this study, we observed inverse associations between serum PLP levels and the risks of cardiovascular and all-cause mortality, and positive associations between 4-PA/PLP and the risks of cardiovascular, cancer, and all-cause mortality. This study suggests that lower serum vitamin B6 levels and higher vitamin B6 turnover rate may be predictors of increased risk of mortality in older adults. Maintaining high levels of vitamin B6 may be beneficial in preventing premature death in the elderly.

## Data availability statement

Publicly available datasets were analyzed in this study. Data can be found here: https://wwwn.cdc.gov/nchs/nhanes/Default.aspx (accessed on 22 March 2022)

## Ethics statement

This study used de-identified, nationally representative survey data that are publicly available upon request to the NHANES. The patients/participants provided their written informed consent to participate in this study.

## Author contributions

PW: Writing – original draft. JH: Writing – review & editing. FX: Methodology, Writing – original draft. MA: Project administration, Writing – review & editing. YT: Project administration, Writing – review & editing. HL: Conceptualization, Methodology, Supervision, Writing – review & editing.

## References

[1] Brunetti G Grugni G Piacente L Delvecchio M Ventura A Giordano P. Analysis of circulating mediators of bone remodeling in prader-willi syndrome. Calcified Tissue Int. (2018) 102:635–43. doi: 10.1007/s00223-017-0376-y 29353451

[2] Barkoukis H. Nutrition recommendations in elderly and aging. Med Clin North Am. (2016) 100:1237–50. doi: 10.1016/j.mcna.2016.06.006 27745592

[3] Norman K Haß U Pirlich M. Malnutrition in older adults-recent advances and remaining challenges. Nutrients. (2021) 13(8):2764. doi: 10.3390/nu13082764 34444924 PMC8399049

[4] Percudani R Peracchi A. The B6 database: A tool for the description and classification of vitamin B6-dependent enzymatic activities and of the corresponding protein families. BMC Bioinf. (2009) 10:273. doi: 10.1186/1471-2105-10-273 PMC2748086 19723314

[5] Stover PJ Field MS. Vitamin B-6. Adv Nutr. (2015) 6:132–3. doi: 10.3945/an.113.005207 PMC4288272 25593152

[6] Ueland PM Ulvik A Rios-Avila L Midttun O Gregory JF. Direct and functional biomarkers of vitamin B6 status. Annu Rev Nutr. (2015) 35:33–70. doi: 10.1146/annurev-nutr-071714-034330 25974692 PMC5988249

[7] Stanger O Herrmann W Pietrzik K Fowler B Geisel J Dierkes J. Dach-liga homocystein (German, Austrian and swiss homocysteine society): consensus paper on the rational clinical use of homocysteine, folic acid and B-vitamins in cardiovascular and thrombotic diseases: guidelines and recommendations. Clin Chem Lab Med. (2003) 41:1392–403. doi: 10.1515/cclm.2003.214 14656016

[8] Stach K Stach W Augoff K. Vitamin B6 in health and disease. Nutrients. (2021) 13. doi: 10.3390/nu13093229 PMC8467949 34579110

[9] Hellmann H Mooney S. Vitamin B6: A molecule for human health? Molecules. (2010) 15:442–59. doi: 10.3390/molecules15010442 PMC6257116 20110903

[10] Minović I Kieneker LM Gansevoort RT Eggersdorfer M Touw DJ Voerman AJ. Vitamin B6, inflammation, and cardiovascular outcome in a population-based cohort: the prevention of renal and vascular end-stage disease (Prevend) study. Nutrients. (2020) 12(9):2711. doi: 10.3390/nu12092711 32899820 PMC7551483

[11] Mocellin S Briarava M Pilati P. Vitamin B6 and cancer risk: A field synopsis and meta-analysis. J Natl Cancer Inst. (2017) 109:1–9. doi: 10.1093/jnci/djw230 28376200

[12] Zhao LG Shu XO Li HL Gao J Han LH Wang J. Prospective cohort studies of dietary vitamin B6 intake and risk of cause-specific mortality. Clin Nutr. (2019) 38:1180–7. doi: 10.1016/j.clnu.2018.04.016 PMC6551204 29764693

[13] Cui R Iso H Date C Kikuchi S Tamakoshi A Japan Collaborative Cohort Study G. Dietary folate and vitamin B6 and B12 intake in relation to mortality from cardiovascular diseases: Japan collaborative cohort study. Stroke. (2010) 41:1285–9. doi: 10.1161/STROKEAHA.110.578906 20395608

[14] Nix WA Zirwes R Bangert V Kaiser RP Schilling M Hostalek U. Vitamin B status in patients with type 2 diabetes mellitus with and without incipient nephropathy. Diabetes Res Clin Pract. (2015) 107:157–65. doi: 10.1016/j.diabres.2014.09.058 25458341

[15] Minović I Riphagen IJ van den Berg E Kootstra-Ros JE van Faassen M Gomes Neto AW. Vitamin B-6 deficiency is common and associated with poor long-term outcome in renal transplant recipients. Am J Clin Nutr. (2017) 105:1344–50. doi: 10.3945/ajcn.116.151431 28468895

[16] Ricci C Freisling H Leitzmann MF Taljaard-Krugell C Jacobs I Kruger HS. Diet and sedentary behaviour in relation to cancer survival. A report from the national health and nutrition examination survey linked to the U.S. Mortality registry. Clin Nutr. (2020) 39:3489–96. doi: 10.1016/j.clnu.2020.03.013 32229168

[17] Zhang D Li Y Lang X Zhang Y. Associations of serum vitamin B6 status and catabolism with all-cause mortality in patients with T2dm. J Clin Endocrinol Metab. (2022) 107:2822–32. doi: 10.1210/clinem/dgac429 PMC9516105 35907182

[18] Bostom AG Carpenter MA Kusek JW Levey AS Hunsicker L Pfeffer MA. Homocysteine-lowering and cardiovascular disease outcomes in kidney transplant recipients: primary results from the folic acid for vascular outcome reduction in transplantation trial. Circulation. (2011) 123:1763–70. doi: 10.1161/CIRCULATIONAHA.110.000588 PMC4887854 21482964

[19] Curtin LR Mohadjer LK Dohrmann SM Montaquila JM Kruszan-Moran D Mirel LB. The national health and nutrition examination survey: sample design, 1999-2006. Vital Health Stat Ser 2 Data Eval Methods Res. (2012) 155):1–39. 22788053

[20] Mirel LB Mohadjer LK Dohrmann SM Clark J Burt VL Johnson CL. National health and nutrition examination survey: estimation procedures, 2007-2010. Vital Health Stat Ser 2 Data Eval Methods Res. (2013) 159):1–17. 25093338

[21] Johnson CL Paulose-Ram R Ogden CL Carroll MD Kruszon-Moran D Dohrmann SM. National health and nutrition examination survey: analytic guidelines, 1999-2010. Vital Health Stat Ser 2 Data Eval Methods Res. (2013) 161):1–24. 25090154

[22] Ulvik A Midttun O Pedersen ER Eussen SJ Nygard O Ueland PM. Evidence for increased catabolism of vitamin B-6 during systemic inflammation. Am J Clin Nutr. (2014) 100:250–5. doi: 10.3945/ajcn.114.083196 24808485

[23] Midttun Ø Hustad S Schneede J Vollset SE Ueland PM. Plasma vitamin B-6 forms and their relation to transsulfuration metabolites in a large, population-based study. Am J Clin Nutr. (2007) 86:131–8. doi: 10.1093/ajcn/86.1.131 17616772

[24] Yang D Liu Y Wang Y Ma Y Bai J Yu C. Association of serum vitamin B6 with all-cause and cause-specific mortality in a prospective study. Nutrients. (2021) 13(9):2977. doi: 10.3390/nu13092977 34578855 PMC8472743

[25] Zhang Z. Multiple imputation with multivariate imputation by chained equation (Mice) package. Ann Transl Med. (2016) 4:30. doi: 10.3978/j.issn.2305-5839.2015.12.63 26889483 PMC4731595

[26] Johansson M Fanidi A Muller DC Bassett JK Midttun O Vollset SE. Circulating biomarkers of one-carbon metabolism in relation to renal cell carcinoma incidence and survival. J Natl Cancer Inst. (2014) 106(12):dju327. doi: 10.1093/jnci/dju327 25376861 PMC4273895

[27] Pusceddu I Herrmann W Kleber ME Scharnagl H Hoffmann MM Winklhofer-Roob BM. Subclinical inflammation, telomere shortening, homocysteine, vitamin B6, and mortality: the ludwigshafen risk and cardiovascular health study. Eur J Nutr. (2020) 59:1399–411. doi: 10.1007/s00394-019-01993-8 PMC7230054 31129702

[28] Schorgg P Karavasiloglou N Beyer A Cantwell M Danquah I Gojda J. Increased vitamin B6 turnover is associated with greater mortality risk in the general us population: A prospective biomarker study. Clin Nutr (Edinburgh Scotland). (2022) 41:1343–56. doi: 10.1016/j.clnu.2022.04.023 35588551

[29] Patterson CC Blankenberg S Ben-Shlomo Y Heslop L Bayer A Lowe G. Which biomarkers are predictive specifically for cardiovascular or for non-cardiovascular mortality in men? Evidence from the caerphilly prospective study (Caps). Int J Cardiol. (2015) 201:113–8. doi: 10.1016/j.ijcard.2015.07.106 PMC4612445 26298350

[30] Camici GG Savarese G Akhmedov A Luscher TF. Molecular mechanism of endothelial and vascular aging: implications for cardiovascular disease. Eur Heart J. (2015) 36:3392–403. doi: 10.1093/eurheartj/ehv587 26543043

[31] McEniery CM Wilkinson IB Avolio AP. Age, hypertension and arterial function. Clin Exp Pharmacol Physiol. (2007) 34:665–71. doi: 10.1111/j.1440-1681.2007.04657.x 17581227

[32] Zaric BL Obradovic M Bajic V Haidara MA Jovanovic M Isenovic ER. Homocysteine and hyperhomocysteinaemia. Curr Med Chem. (2019) 26:2948–61. doi: 10.2174/0929867325666180313105949 29532755

[33] Ulvik A Pedersen ER Svingen GF McCann A Midttun O Nygard O. Vitamin B-6 catabolism and long-term mortality risk in patients with coronary artery disease. Am J Clin Nutr. (2016) 103:1417–25. doi: 10.3945/ajcn.115.126342 27169836

[34] Zuo H Ueland PM Eussen SJ Tell GS Vollset SE Nygård O. Markers of vitamin B6 status and metabolism as predictors of incident cancer: the hordaland health study. Int J Cancer. (2015) 136:2932–9. doi: 10.1002/ijc.29345 25404109

[35] Cui Q Zhu X Guan G Hui R Zhu L Wang J. Associations of vitamin B6 turnover rate with the risk of cardiovascular and all-cause mortality in hypertensive adults. Nutr Metab Cardiovasc Dis. (2023) 33:1225–34. doi: 10.1016/j.numecd.2023.03.017 37085414

[36] Talbott MC Miller LT Kerkvliet NI. Pyridoxine supplementation: effect on lymphocyte responses in elderly persons. Am J Clin Nutr. (1987) 46:659–64. doi: 10.1093/ajcn/46.4.659 3499066

[37] Casciato DA McAdam LP Kopple JD Bluestone R Goldberg LS Clements PJ. Immunologic abnormalities in hemodialysis patients: improvement after pyridoxine therapy. Nephron. (1984) 38:9–16. doi: 10.1159/000183270 6472538

[38] Cheng CH Chang SJ Lee BJ Lin KL Huang YC. Vitamin B6 supplementation increases immune responses in critically ill patients. Eur J Clin Nutr. (2006) 60:1207–13. doi: 10.1038/sj.ejcn.1602439 16670691

[39] Meydani SN Ribaya-Mercado JD Russell RM Sahyoun N Morrow FD Gershoff SN. Vitamin B-6 deficiency impairs interleukin 2 production and lymphocyte proliferation in elderly adults. Am J Clin Nutr. (1991) 53:1275–80. doi: 10.1093/ajcn/53.5.1275 2021134

[40] Huang YC Chang HH Huang SC Cheng CH Lee BJ Cheng SY. Plasma pyridoxal 5’-phosphate is a significant indicator of immune responses in the mechanically ventilated critically ill. Nutrition. (2005) 21:779–85. doi: 10.1016/j.nut.2004.11.013 15975484

[41] Ulvik A Midttun O Pedersen ER Nygard O Ueland PM. Association of Plasma B-6 Vitamers with Systemic Markers of Inflammation before and after Pyridoxine Treatment in Patients with Stable Angina Pectoris. Am J Clin Nutr. (2012) 95:1072–8. doi: 10.3945/ajcn.111.029751 22492365

[42] Ueland PM McCann A Midttun O Ulvik A. Inflammation, vitamin B6 and related pathways. Mol Aspects Med. (2017) 53:10–27. doi: 10.1016/j.mam.2016.08.001 27593095

[43] Chiang EP Smith DE Selhub J Dallal G Wang YC Roubenoff R. Inflammation causes tissue-specific depletion of vitamin B6. Arthritis Res Ther. (2005) 7:R1254–62. doi: 10.1186/ar1821 PMC1297572 16277678

[44] Ridker PM Everett BM Thuren T MacFadyen JG Chang WH Ballantyne C. Antiinflammatory therapy with canakinumab for atherosclerotic disease. N Engl J Med. (2017) 377:1119–31. doi: 10.1056/NEJMoa1707914 28845751

[45] Hansson GK Libby P. The immune response in atherosclerosis: A double-edged sword. Nat Rev Immunol. (2006) 6:508–19. doi: 10.1038/nri1882 16778830

[46] Elinav E Nowarski R Thaiss CA Hu B Jin C Flavell RA. Inflammation-induced cancer: crosstalk between tumours, immune cells and microorganisms. Nat Rev Cancer. (2013) 13:759–71. doi: 10.1038/nrc3611 24154716

[47] Colotta F Allavena P Sica A Garlanda C Mantovani A. Cancer-related inflammation, the seventh hallmark of cancer: links to genetic instability. Carcinogenesis. (2009) 30:1073–81. doi: 10.1093/carcin/bgp127 19468060

[48] Federico A Morgillo F Tuccillo C Ciardiello F Loguercio C. Chronic inflammation and oxidative stress in human carcinogenesis. Int J Cancer. (2007) 121:2381–6. doi: 10.1002/ijc.23192 17893868

