# Supplementary material for: Associations of serum vitamin B6 status with the risks of cardiovascular, cancer, and all-cause mortality in the elderly
Source: Front Immunol. 2024 Apr 18;15:1354958. doi: 10.3389/fimmu.2024.1354958 (PMC11064647; doi:10.3389/fimmu.2024.1354958)
Supplement: Supplementary file 1 [file Table_1.docx]

Supplementary Material

Associations of serum vitamin B6 status with the risks of cardiovascular, cancer, and all-cause mortality in the elderly

**Pengxi Wang*****,** **Jia Huang*****,** **Feng Xu,** **Munire Abuduaini,** **Yuchang Tao**

*** Correspondence:** Hongyan Liu: [liuhongyanqhhy@126.com](mailto:liuhongyanqhhy@126.com)

# Supplementary Tables

**Table S1.** Stratified analyses of the associations between vitamin B6 biomarker levels and cardiovascular disease (CVD) mortality among the U.S. older adults

|  | Quartile of vitamin B6 biomarkers | | | |  | | P _interaction_ |
| --- | --- | --- | --- | --- | --- | --- | --- |
|  | Q1 | Q2 | Q3 | Q4 | | P _trend_ |  |
| **PLP, nmol/L** |  |  |  |  | |  |  |
| Age |  |  |  |  | |  | 0.056 |
| <70 | Ref | 1.47 (0.77-2.82) | 1.09(0.54-2.23) | 0.64(0.27-1.54) | | 0.149 |  |
| ≥70 | Ref | 0.72(0.53-0.98) | 0.76(0.53-1.09) | 0.70(0.54-0.91) | | 0.059 |  |
| Sex |  |  |  |  | |  | **0.025** |
| male | Ref | 1.00(0.67-1.49) | 0.81(0.52-1.26) | 0.54(0.37-0.78) | | <0.001 |  |
| female | Ref | 0.65(0.43-1.00) | 0.73(0.44-1.19) | 0.84(0.54-1.30) | | 0.194 |  |
| BMI, kg/m^2^ |  |  |  |  | |  | 0.697 |
| <30 | Ref | 0.76(0.56-1.05) | 0.79(0.57-1.10) | 0.66(0.48-0.92) | | 0.085 |  |
| ≥30 | Ref | 0.93(0.58-1.49) | 0.71(0.42-1.21) | 0.77(0.48-1.22) | | 0.418 |  |
| Smoke |  |  |  |  | |  | **0.043** |
| never | Ref | 1.06(0.69-1.63) | 1.07(0.69-1.66) | 0.69(0.43-1.11) | | 0.107 |  |
| former or current | Ref | 0.68(0.45-1.01) | 0.59(0.38-0.90) | 0.73(0.51-1.06) | | 0.086 |  |
| Drink |  |  |  |  | |  | 0.494 |
| no | Ref | 0.95(0.58-1.55) | 1.05(0.63-1.76) | 0.81(0.49-1.36) | | 0.612 |  |
| yes | Ref | 0.77(0.54-1.10) | 0.63(0.42-0.92) | 0.63(0.47-0.84) | | 0.003 |  |
| **4-PA, nmol/L** |  |  |  |  | |  |  |
| Age |  |  |  |  | |  | 0.491 |
| <70 | Ref | 1.52(0.74-3.11) | 1.20(0.50-2.88) | 1.51(0.66-3.42) | | 0.693 |  |
| ≥70 | Ref | 1.14(0.70-1.85) | 1.42(0.89-2.24) | 1.55(1.02-2.34) | | 0.065 |  |
| Sex |  |  |  |  | |  | 0.316 |
| male | Ref | 1.23(0.68-2.25) | 1.37(0.76-2.47) | 1.22(0.70-2.11) | | 0.720 |  |
| female | Ref | 1.15(0.64-2.07) | 1.14(0.61-2.13) | 1.46(0.85-2.51) | | 0.164 |  |
| BMI, kg/m^2^ |  |  |  |  | |  | 0.438 |
| <30 | Ref | 1.10(0.65-1.85) | 1.23(0.72-2.11) | 1.17(0.73-1.88) | | 0.862 |  |
| ≥30 | Ref | 1.18(0.66-2.13) | 1.21(0.65-2.25) | 1.67(1.01-2.75) | | 0.063 |  |
| Smoke |  |  |  |  | |  | 0.614 |
| never | Ref | 1.24(0.76-2.04) | 1.53(0.88-2.62) | 1.42(0.84-2.40) | | 0.495 |  |
| former or current | Ref | 1.08(0.62-1.91) | 1.07(0.60-1.89) | 1.30(0.80-2.12) | | 0.359 |  |
| Drink |  |  |  |  | |  | 0.232 |
| no | Ref | 1.17(0.72-1.89) | 1.66(0.98-2.82) | 1.29(0.73-2.27) | | 0.231 |  |
| yes | Ref | 1.13(0.64-2.01) | 1.05(0.57-1.92) | 1.37(0.84-2.24) | | 0.202 |  |
| **Ratio 4-PA/ PLP** |  |  |  |  | |  |  |
| Age |  |  |  |  | |  | 0.512 |
| <70 | Ref | 2.21(0.84-5.82) | 1.87(0.86-4.06) | 2.90(1.09-7.72) | | 0.208 |  |
| ≥70 | Ref | 0.95(0.63-1.44) | 1.40(1.01-1.94) | 2.09(1.58-2.76) | | <0.001 |  |
| Sex |  |  |  |  | |  | 0.703 |
| male | Ref | 1.30(0.78-2.17) | 1.85(1.24-2.76) | 2.44(1.50-3.96) | | <0.001 |  |
| female | Ref | 1.09(0.57-2.08) | 1.16(0.66-2.07) | 1.67(0.99-2.83) | | 0.063 |  |
| BMI, kg/m^2^ |  |  |  |  | |  | 0.465 |
| <30 | Ref | 1.29(0.83-1.99) | 1.67(1.12-2.50) | 1.96(1.28-3.00) | | 0.010 |  |
| ≥30 | Ref | 1.06(0.57-1.99) | 1.22(0.76-1.95) | 2.09(1.25-3.50) | | 0.008 |  |
| Smoke |  |  |  |  | |  | 0.497 |
| never | Ref | 0.99(0.54-1.81) | 1.32(0.79-2.21) | 1.59(1.00-2.54) | | 0.089 |  |
| former or current | Ref | 1.50(0.95-2.36) | 1.71(1.12-2.61) | 2.52(1.60-4.00) | | <0.001 |  |
| Drink |  |  |  |  | |  | 0.387 |
| no | Ref | 1.28(0.66-2.51) | 1.28(0.67-2.45) | 1.53(0.83-2.84) | | 0.543 |  |
| yes | Ref | 1.13(0.73-1.76) | 1.64(1.14-2.34) | 2.37(1.59-3.53) | | <0.001 |  |

adjusted for age (not for age stratiﬁed analysis), sex (not for sex stratiﬁed analysis), smoke (not for smoke stratiﬁed analysis), drink (not for drink stratiﬁed analysis), race/ethnicity, education, activity, and BMI (not for BMI stratiﬁed analysis).

Abbreviations: BMI, body mass index; PLP, pyridoxal 5'-phosphate; 4-PA, 4-pyridoxic acid.

**Table S2.** Stratified analyses of the associations between vitamin B6 biomarker levels and cancer mortality among the U.S. older adults

|  | Quartile of vitamin B6 biomarkers | | | |  | P _interaction_ |
| --- | --- | --- | --- | --- | --- | --- |
|  | Q1 | Q2 | Q3 | Q4 | P _trend_ |  |
| **PLP, nmol/L** |  |  |  |  |  |  |
| Age |  |  |  |  |  | 0.132 |
| <70 | Ref | 1.14(0.71-1.83) | 0.98(0.56-1.73) | 0.50(0.25-1.00) | 0.127 |  |
| ≥70 | Ref | 1.12(0.75-1.66) | 0.82(0.54-1.25) | 0.86(0.57-1.30) | 0.312 |  |
| Sex |  |  |  |  |  | **0.030** |
| male | Ref | 0.74 (0.49-1.11) | 0.75(0.48-1.16) | 0.67(0.40-1.12) | 0.425 |  |
| female | Ref | 1.92(1.12-3.31) | 1.11(0.64-1.91) | 0.84(0.44-1.62) | 0.003 |  |
| BMI, kg/m^2^ |  |  |  |  |  | 0.052 |
| <30 | Ref | 1.08(0.73-1.60) | 0.66(0.41-1.06) | 0.73(0.48-1.13) | 0.071 |  |
| ≥30 | Ref | 1.20(0.77-1.85) | 1.34(0.87-2.07) | 0.69(0.37-1.27) | 0.108 |  |
| Smoke |  |  |  |  |  | 0.194 |
| never | Ref | 1.29(0.67-2.52) | 1.30(0.70-1.41) | 0.66(0.38-1.13) | 0.083 |  |
| former or current | Ref | 1.09(0.78-1.54) | 0.75(0.48-1.17) | 0.81(0.53-1.22) | 0.172 |  |
| Drink |  |  |  |  |  | 0.548 |
| no | Ref | 1.37(0.81-2.31) | 1.15(0.66-2.00) | 0.75(0.43-1.28) | 0.174 |  |
| yes | Ref | 1.03(0.70-1.52) | 0.79(0.53-1.17) | 0.70(0.45-1.09) | 0.109 |  |
| **4-PA, nmol/L** |  |  |  |  |  |  |
| Age |  |  |  |  |  | 0.404 |
| <70 | Ref | 1.29(0.82-2.03) | 0.98(0.50-1.91) | 1.06(0.52-2.15) | 0.560 |  |
| ≥70 | Ref | 0.98(0.61-1.56) | 1.07(0.68-1.69) | 1.13(0.69-1.85) | 0.906 |  |
| Sex |  |  |  |  |  | 0.582 |
| male | Ref | 0.90(0.62-1.30) | 1.05(0.71-1.55) | 1.25(0.79-1.97) | 0.640 |  |
| female | Ref | 1.31(0.72-2.38) | 0.98(0.54-1.78) | 0.82(0.43-1.56) | 0.206 |  |
| BMI, kg/m^2^ |  |  |  |  |  | 0.847 |
| <30 | Ref | 0.98(0.62-1.55) | 1.06(0.68-1.65) | 1.02(0.57-1.81) | 0.987 |  |
| ≥30 | Ref | 1.16(0.68-1.97) | 0.85(0.44-1.65) | 1.12(0.64-1.97) | 0.792 |  |
| Smoke |  |  |  |  |  | **0.002** |
| never | Ref | 1.74(0.91-3.29) | 2.24(1.19-4.19) | 1.25(0.65-2.40) | 0.038 |  |
| former or current | Ref | 0.93(0.64-1.35) | 0.75(0.50-1.14) | 1.08(0.66-1.76) | 0.177 |  |
| Drink |  |  |  |  |  | 0.517 |
| no | Ref | 1.10(0.58-2.08) | 1.06(0.57-1.96) | 0.77(0.39-1.53) | 0.771 |  |
| yes | Ref | 0.99(0.66-1.47) | 0.96(0.66-1.39) | 1.14(0.73-1.80) | 0.780 |  |
| **Ratio 4-PA/ PLP** |  |  |  |  |  |  |
| Age |  |  |  |  |  | **0.014** |
| <70 | Ref | 2.33(1.14-4.76) | 2.15(1.05-4.43) | 2.82(1.25-6.34) | 0.074 |  |
| ≥70 | Ref | 0.71(0.49-1.02) | 0.94(0.59-1.50) | 1.50(0.98-2.28) | <0.001 |  |
| Sex |  |  |  |  |  | **0.009** |
| male | Ref | 1.71(1.08-2.72) | 1.87(1.05-3.32) | 3.07(1.79-5.25) | <0.001 |  |
| female | Ref | 0.88(0.52-1.50) | 0.94(0.46-1.95) | 1.12(0.67-1.86) | 0.706 |  |
| BMI, kg/m^2^ |  |  |  |  |  | 0.252 |
| <30 | Ref | 1.58(0.93-2.68) | 1.68(0.97-2.92) | 2.22(1.33-3.72) | 0.016 |  |
| ≥30 | Ref | 0.84(0.44-1.62) | 0.94(0.51-1.72) | 1.49(0.79-2.81) | 0.117 |  |
| Smoke |  |  |  |  |  | 0.881 |
| never | Ref | 1.09(0.58-2.04) | 1.16(0.64-2.10) | 1.59(0.84-3.00) | 0.422 |  |
| former or current | Ref | 1.35(0.83-2.19) | 1.44(0.82-2.54) | 2.13(1.28-3.53) | 0.003 |  |
| Drink |  |  |  |  |  | **0.007** |
| no | Ref | 0.83(0.41-1.68) | 0.81(0.36-1.83) | 0.85(0.41-1.78) | 0.949 |  |
| yes | Ref | 1.50(0.96-2.34) | 1.66(1.02-2.71) | 2.65(1.69- 4.17) | <0.001 |  |

adjusted for age (not for age stratiﬁed analysis), sex (not for sex stratiﬁed analysis), smoke (not for smoke stratiﬁed analysis), drink (not for drink stratiﬁed analysis), race/ethnicity, education, activity, and BMI (not for BMI stratiﬁed analysis).

Abbreviations: BMI, body mass index; PLP, pyridoxal 5'-phosphate; 4-PA, 4-pyridoxic acid.

**Table S3.** Stratified analyses of the associations between vitamin B6 biomarker levels and all-cause mortality among the U.S. older adults

|  | Quartile of vitamin B6 biomarkers | | | |  | P _interaction_ |
| --- | --- | --- | --- | --- | --- | --- |
|  | Q1 | Q2 | Q3 | Q4 | P _trend_ |  |
| **PLP, nmol/L** |  |  |  |  |  |  |
| Age |  |  |  |  |  | **0.006** |
| <70 | Ref | 0.91(0.64-1.30) | 0.76(0.57-1.03) | 0.49(0.33-0.71) | <0.001 |  |
| ≥70 | Ref | 0.82(0.71-0.96) | 0.71(0.59-0.84) | 0.68(0.58-0.81) | <0.001 |  |
| Sex |  |  |  |  |  | 0.872 |
| male | Ref | 0.80 (0.63-1.01) | 0.70(0.58-0.85) | 0.61(0.47-0.78) | <0.001 |  |
| female | Ref | 0.82(0.64-1.04) | 0.64(0.50-0.81) | 0.62(0.47-0.81) | <0.001 |  |
| BMI, kg/m^2^ |  |  |  |  |  | 0.946 |
| <30 | Ref | 0.77(0.64-0.92) | 0.60(0.51-0.71) | 0.61(0.51-0.73) | <0.001 |  |
| ≥30 | Ref | 0.92(0.72-1.17) | 0.87(0.70-1.09) | 0.63(0.49-0.82) | 0.003 |  |
| Smoke |  |  |  |  |  | 0.212 |
| never | Ref | 0.83(0.67-1.02) | 0.79(0.61-1.04) | 0.63(0.47-0.84) | 0.008 |  |
| former or current | Ref | 0.84(0.69-1.02) | 0.62(0.51-0.75) | 0.64(0.50-0.81) | <0.001 |  |
| Drink |  |  |  |  |  | 0.360 |
| no | Ref | 0.95(0.74-1.21) | 0.83(0.64- 1.07) | 0.66(0.50-0.87) | 0.022 |  |
| yes | Ref | 0.76(0.63-0.90) | 0.60(0.51-0.72) | 0.60(0.51-0.70) | <0.001 |  |
| **4-PA, nmol/L** |  |  |  |  |  |  |
| Age |  |  |  |  |  | 0.062 |
| <70 | Ref | 1.00(0.71-1.41) | 1.19(1.89-1.61) | 0.99(0.66-1.49) | 0.359 |  |
| ≥70 | Ref | 0.99(0.83-1.19) | 1.19(0.99-1.43) | 1.27(1.00-1.60) | 0.040 |  |
| Sex |  |  |  |  |  | 0.604 |
| male | Ref | 0.98(0.80-1.20) | 1.18(0.93-1.50) | 1.19(0.94-1.50) | 0.198 |  |
| female | Ref | 0.86(0.66-1.11) | 0.98(0.77-1.24) | 0.93(0.68-1.27) | 0.541 |  |
| BMI, kg/m^2^ |  |  |  |  |  | 0.228 |
| <30 | Ref | 0.87(0.71-1.08) | 1.05(0.84-1.32) | 1.01(0.78-1.29) | 0.296 |  |
| ≥30 | Ref | 0.98(0.70-1.37) | 1.06(0.80-1.41) | 1.15(0.84-1.57) | 0.614 |  |
| Smoke |  |  |  |  |  | 0.338 |
| never | Ref | 0.99(0.73-1.36) | 1.16(0.82-1.65) | 1.01(0.68-1.49) | 0.484 |  |
| former or current | Ref | 0.88(0.71-1.10) | 1.03(0.82-1.31) | 1.12(0.85-1.47) | 0.110 |  |
| Drink |  |  |  |  |  | 0.360 |
| no | Ref | 0.83(0.62-1.12) | 1.05(0.78-1.40) | 0.90(0.64-1.28) | 0.198 |  |
| yes | Ref | 0.98(0.77-1.25) | 1.09(0.84-1.42) | 1.15(0.88-1.49) | 0.409 |  |
| **Ratio 4-PA/ PLP** |  |  |  |  |  |  |
| Age |  |  |  |  |  | 0.410 |
| <70 | Ref | 1.67(1.18-2.35) | 1.93(1.31-2.85) | 2.56(1.71-3.85) | <0.001 |  |
| ≥70 | Ref | 1.22(0.93-1.59) | 1.54(1.24-1.92) | 2.38(1.93-2.93) | <0.001 |  |
| Sex |  |  |  |  |  | 0.175 |
| male | Ref | 1.44(1.12-1.86) | 1.86(1.43-2.42) | 2.83(2.16-3.71) | <0.001 |  |
| female | Ref | 1.32(0.96-1.82) | 1.52(1.21-1.92) | 1.92(1.46-2.54) | <0.001 |  |
| BMI, kg/m^2^ |  |  |  |  |  | 0.555 |
| <30 | Ref | 1.59(1.20-2.12) | 1.87(1.45-2.40) | 2.47(1.94-3.15) | <0.001 |  |
| ≥30 | Ref | 1.04(0.77-1.41) | 1.31(0.99-1.72) | 1.92(1.38-2.68) | <0.001 |  |
| Smoke |  |  |  |  |  | 0.108 |
| never | Ref | 1.26(0.94-1.69) | 1.65(01.26-2.14) | 1.83(1.40-2.41) | <0.001 |  |
| former or current | Ref | 1.47(1.16-1.87) | 1.64(1.26-2.14) | 2.59(2.03-3.31) | <0.001 |  |
| Drink |  |  |  |  |  | 0.360 |
| no | Ref | 1.28(0.83-1.99) | 1.43(1.00-2.05) | 1.83(1.30-2.58) | <0.001 |  |
| yes | Ref | 1.43(1.17-1.74) | 1.79(1.40-2.30) | 2.56(1.96-3.34) | <0.001 |  |

adjusted for age (not for age stratiﬁed analysis), sex (not for sex stratiﬁed analysis), smoke (not for smoke stratiﬁed analysis), drink (not for drink stratiﬁed analysis), race/ethnicity, education, activity, and BMI (not for BMI stratiﬁed analysis).

Abbreviations: BMI, body mass index; PLP, pyridoxal 5'-phosphate; 4-PA, 4-pyridoxic acid.
